# TPO as an indicator of lymph node metastasis and recurrence in papillary thyroid carcinoma

**DOI:** 10.1038/s41598-023-37932-1

**Published:** 2023-07-05

**Authors:** Xiang Li, Ruochuan Cheng

**Affiliations:** Department of Thyroid Surgery, The First Affiliated Hospital of Kunming Medical University, No. 295, Xichang Road, Kunming, 650032 Yunnan China

**Keywords:** Head and neck cancer, Metastasis, Tumour biomarkers, Tumour immunology

## Abstract

The objective of this study was to investigate the expression of thyroid peroxidase (TPO) in papillary thyroid carcinoma (PTC) and to preliminarily investigate its value as a marker of lymph node metastasis and recurrence in patients with PTC. Clinical data of PTC patients and TPO expression were collected from The Cancer Genome Atlas (TCGA) database for analysis. We recruited 230 consecutive PTC patients from the Department of Thyroid Surgery of the First Affiliated Hospital of Kunming Medical University, collected their clinicopathological data, and also performed immunohistochemical analysis of TPO expression on their thyroid specimens to validate the results of bioinformatics analysis. In addition, the construction of protein–protein interaction networks was performed too. Functional enrichment analysis and immuno-infiltration analysis characterized the pathways in which TPO genes may be involved. Data mining based on the TCGA database showed that TPO expression in PTC tissues was significantly lower than in paired normal thyroid tissues. The expression level of TPO in PTC tissues correlated with tumor lymph node metastasis and recurrence. Follow-up data from our center also validated the difference in TPO expression and its relationship with lymph node metastasis in PTC patients. Functional enrichment analysis showed that TPO function was significantly associated with signaling pathways related to amino acid metabolism, gene expression regulation and tumorigenesis. TPO expression was also significantly associated with immune infiltration. Our study showed that reduced TPO expression was significantly associated with lymph node metastasis and recurrence in patients with PTC, and we validated this result in our central cohort. These data suggest that TPO may serve as a prognostic indicator for PTC.

## Introduction

Thyroid cancer (TC) is the most common endocrine tumor, and its incidence is growing^1^. Thyroid cancer can occur in all age groups, but is predominant in young and middle-aged women^2^. Pathologically, thyroid cancer can be divided into papillary thyroid carcinoma, follicular thyroid carcinoma, medullary thyroid carcinoma, and undifferentiated thyroid carcinoma. Of these, papillary thyroid carcinoma is the most common type, accounting for approximately 80–90% of new thyroid cancers^3,4^. Papillary thyroid carcinoma has a good prognosis^5^, but is prone to early lymph node metastasis^6^. It has also been shown that there is a strong relationship between lymph node metastasis and disease recurrence in PTC^7,8^. The recurrence rate of PTC patients reaches 25% according to long-term follow-up statistics^9^. Therefore, early prediction of lymph node metastasis is particularly important for the prognosis of patients.

The human peroxidase gene, located at 2p25, is approximately 150 Kb long and consists of 16 introns and 17 exons. The mRNA encoding the thyroid-specific protein thyroid peroxidase (TPO) is 3152 bp long and the full length of the CDS is 2802bp^10^. TPO has an important role in thyroid hormone synthesis and maintenance of stable thyroid function. According to previous reports, TPO is mostly highly expressed in normal thyroid groups and benign thyroid disease, while it is mostly low or absent in thyroid cancer^11,12^. Therefore, TPO expression is often used clinically to assist in the diagnosis of benign and malignant thyroid tumors with high sensitivity and specificity^13^. However, there are still some patients with PTC whose thyroid cancer tissues show positive expression of TPO.

There is a lack of reliable clinical indicators to predict lymph node metastasis and recurrence of PTC. It is important to find predictive biomarkers associated with lymph node metastasis and recurrence in PTC, which is important to guide clinical treatment of PTC. In our clinical observation, we found that the rate of lymph node metastasis and recurrence in patients with positive TPO expression in PTC tumor tissues may be better than those with negative TPO expression, so TPO may be used as a predictor of lymph node metastasis as well as tumor recurrence.

To investigate the potential value of differential TPO in PTC patients, this study assessed the relationship between TPO expression and PTC lymph node metastasis and tumor recurrence using data obtained from the TCGA dataset, using specimens from PTC patients at our center and clinicopathological data used to validate the results of bioinformatics analysis. We also analyzed TPO-related signaling pathways that may be associated with PTC progression and prognosis. Our findings may reveal potential therapeutic targets and provide insights into the molecular mechanisms of PTC.

## Materials and methods

### Analysis of TPO expression and clinical significance based on TCGA and HPA databases

We retrieved mRNA expression and clinical information data of human thyroid cancer based on data mining in the TCGA database. Combining the immunohistochemical data of thyroid cancer tissues and normal tissues in the Human Protein Atlas database, we first compared the expression of TPO in thyroid cancer tissues and normal thyroid tissues. We then extracted the clinical data and prognostic information of PTC patients from all available samples and analyzed the relationship between their TPO expression for PTC lymph node metastasis and recurrence in conjunction with their TPO expression. This process is carried out through the “ggplot2” software package in R^14^.

### Validation of TPO expression and role in PTC based on data from PTC patients in our center

We recruited 230 consecutive patients with PTC who were hospitalized in the Department of Thyroid Surgery of the First Affiliated Hospital of Kunming Medical University for surgical treatment, collected their thyroid specimens and clinicopathological data, and performed immunohistochemical analysis of the patients’ PTC tissues and their normal tissues to verify the difference in TPO expression between PTC tissues and normal thyroid tissues.

All specimen tissues were sectioned into 4 µm serial sections. After dewaxing, hydration, antigen repair, and goat serum blocking, the sections were incubated with primary murine anti-human TPO polyclonal antibody at 4 °C overnight. Then the sections were washed with sterile phosphate-buffered saline (PBS) and incubated with secondary antibodies for 1 h at room temperature. After adding substrate and hematoxylin staining, the slides were observed under a microscope. The immunohistochemical results were independently recorded and reviewed by two experienced pathologists. If brownish yellow or brown particles are seen in the cytoplasm or cell membrane, they are labeled as positive. And if the cells are not stained, they are labeled as negative.

The effect of TPO expression on lymph node metastasis in PTC patients was also verified in conjunction with the clinicopathological data of the patients.

### Protein–protein interaction (PPI) network construction and prediction of signaling pathways

The Search Tool for Retrieval of Interacting Genes (STRING) database is a database for predicting functional associations between proteins that contains 261,033 direct homologs from 89 fully sequenced genomes^15^. The results were visualized using Cytoscape software.

### TPO expression-based enrichment pathway analysis

To explore the possible molecular mechanisms behind key genes, we use Gene Ontology (GO) terminology and Kyoto Encyclopedia of Genes and Genomes (KEGG) analysis for identifying important pathways. This process is carried out through the ‘clusterProfiler’ software package in R^16^. GO functional analysis is used to investigate the function of large-scale genomic or transcriptomic data, including biological processes (BP), cellular components (CC), and molecular functions (MF). Of these, BP is used to explain the biological processes in which genes are involved, CC is used to explain where genes are present, and MF is used to explain the function of genes at the molecular level. KEGG is an annotation of the function of the gene itself, analyzing the various pathways in which the gene is involved. To further enrich the biological functions and pathways between high and low risk groups, we assessed important common pathways of gene enrichment by gene set enrichment analysis (GSEA). Samples were divided into high and low TPO groups as training sets to distinguish potential functions and elucidate significant survival differences using GSEA. We compared pathways with TPO enrichment in each phenotype using nominal *p* values and normalized enrichment scores (NES).

### Immune infiltration analysis

The 24 immune cell markers were extracted from the study of Bindea and colleagues^17^. The ssGSEA method was used to analyze the infiltration of 24 immune cells in the tumor and Spearman correlation was used to analyze the correlation of TPO with these 24 immune cells^18^.

### Statistical analysis

Statistical analyses were performed using R software (version.3.6.1; https://www.r-project.org/). Chi-squared test and t-test were used to verify the relationship between clinicopathological characteristics and TPO, then the variables that might be meaningful in the univariate analysis were included in the multivariate analysis, and the significance was verified by logistic regression analysis. *P* < 0.05 is considered statistically significant.

### Ethics approval

The content and process of this study have been reviewed and approved by the University Ethics Committee of the First Affiliated Hospital of Kunming Medical University in accordance with international and national ethical requirements. In compliance with the Declaration of Helsinki, patients gave their informed consent before material was obtained for use in the study.

## Results

### Differential expression of TPO between thyroid cancer and normal tissue

**Figure 1:**
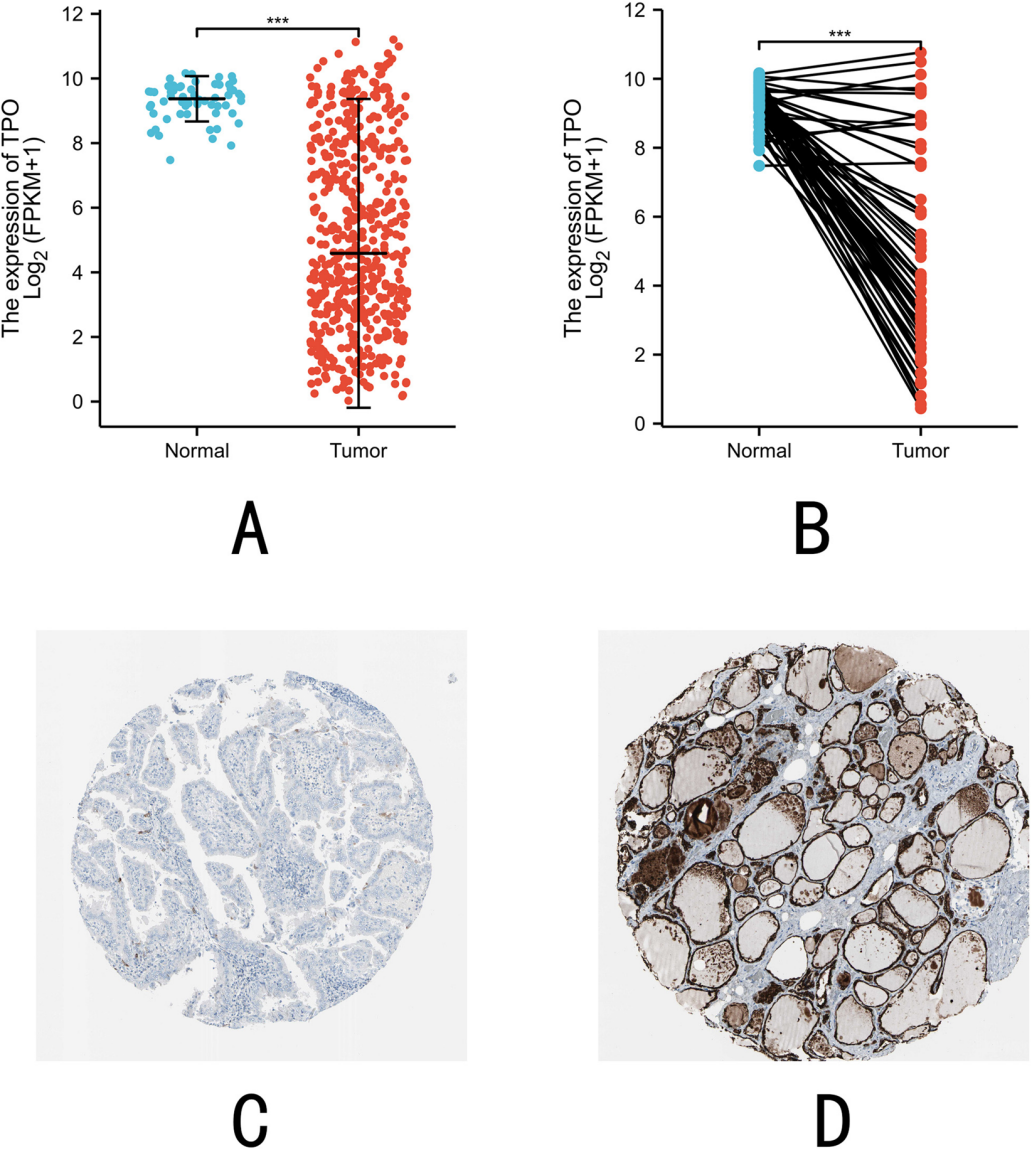
Expression levels of TPO in thyroid cancer and normal thyroid tissues. (**A**) The mRNA expression of TPO in TC samples and unpaired normal thyroid tissue samples. (**B**) mRNA expression of TPO in TC samples and paired normal thyroid tissue samples. Immunohistochemical results from the HPA database show (**C**) Negative expression of TPO in normal thyroid tissues. (**D**) Positive expression of TPO in PTC tissues.

We analyzed data from the TCGA database to compare the expression levels of TPO between thyroid cancer and normal thyroid tissue. We examined its expression using the RNA-seq data in TCGA-THCA. In this cohort, RNA-seq was performed on 510 primary TC tissues and 58 normal thyroid tissues. Scatter plots show the difference in TPO expression between normal and tumor samples (*P* < 0.001, Fig. 1A). Paired plots were used to show the difference in TPO expression between normal and tumor samples from the same patient and showed a significant difference (*P* < 0.001, Fig. 1B). The findings suggest that TPO expression is significant and may play an important role in regulating cancer development.

Subsequently, we used the results of IHC staining of human tissues from the HPA (Human Protein Atlas) database to compare TPO protein expression in PTC tissues and normal thyroid tissues. The staining results showed that TPO expression in PTC tissues was often negative (Fig. 1C). In contrast, TPO expression in normal thyroid tissues was often positive (Fig. 1D). These findings suggest that TPO is downregulated at both the RNA and protein levels in PTC compared with normal thyroid tissues.

### Relationship between clinicopathological factors and TPO gene expression

**Figure 2:**
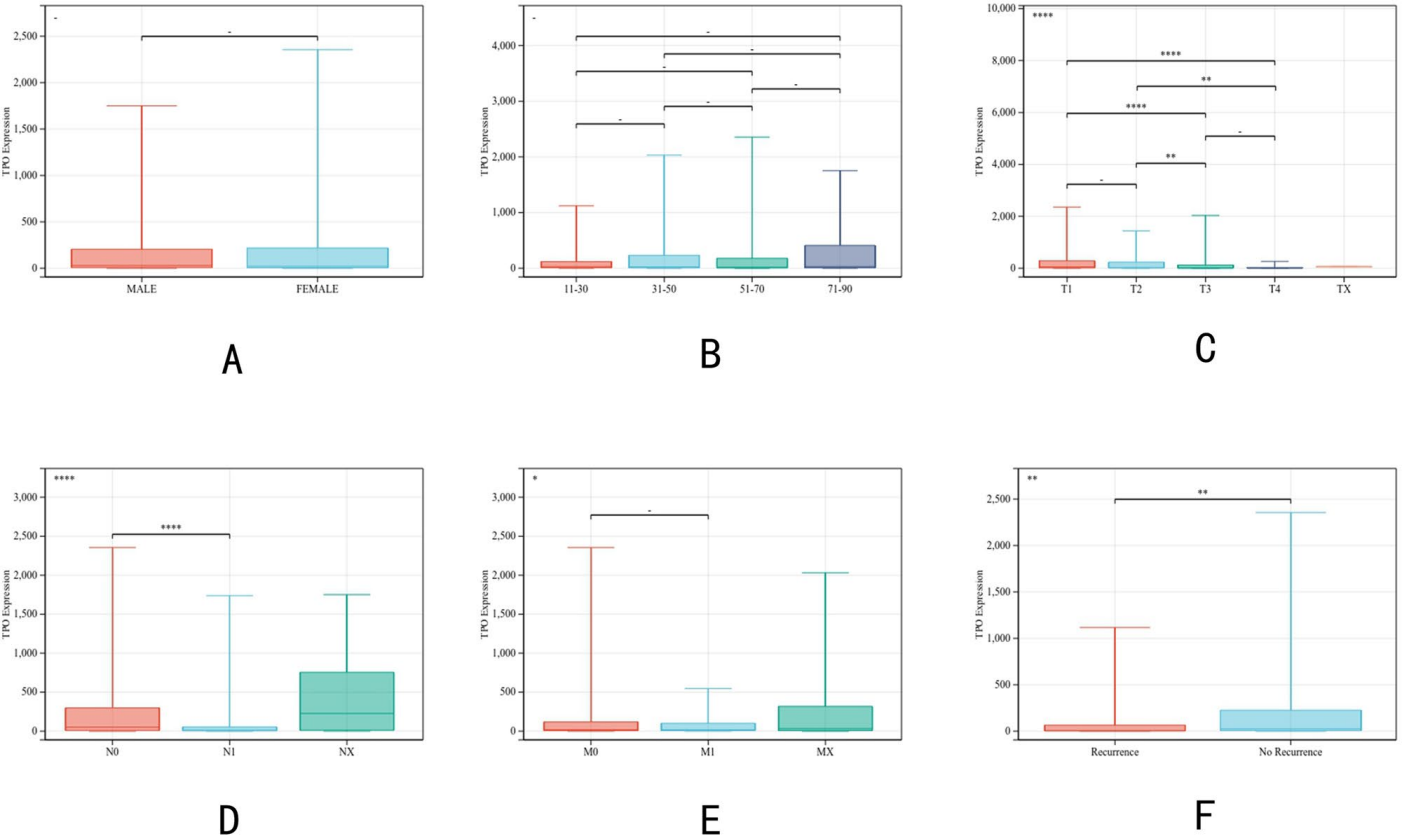
Relationship between transcript expression of TPO from the TCGA cohort and clinicopathological parameters of PTC patients. (**A**) Relationship between transcript expression of TPO and patient gender. (**B**) Relationship between transcript expression of TPO and patient age. (**C**) Relationship between transcript expression of TPO and tumor grade of patients. (**D**) Relationship between transcript expression of TPO and tumor T-stage of patients. (**E**) Relationship between the transcriptional expression of TPO and the N-stage of patients’ tumors. (**F**) Relationship between the transcriptional expression of TPO and the M-stage of patients’ tumors.

TPO gene expression correlated with the clinicopathological characteristics of PTC in the TCGA dataset (Fig. 2A–F). First, we explored the relationship between TPO expression and patients’ age and gender, and the results showed that there was no significant correlation between TPO expression and patients’ age and gender. We then explored the expression of TPO at different tumor stages. The results showed that TPO was significantly higher in stage IV than in stages I and II (*p* < 0.0001). Finally, we also compared the association between TPO expression and T, N, and M stages of PTC. The results confirmed that TPO expression differed significantly in T and N stages, and the analysis showed that TPO expression levels were significantly lower in T3 and T4 stages than in T1 stage. TPO expression was significantly higher in PTC patients without lymph node metastasis than in PTC patients with lymph node metastasis (*p* < 0.0001). And TPO expression did not differ between M0 and M1 stages.

### Association of TPO expression with PTC lymph node metastasis and recurrence

To assess the association of TPO expression with lymph node metastasis and recurrence in patients, we obtained transcriptional expression data and clinicopathological data from TCGA and derived the results by multifactorial analysis. logistic regression analysis of lymph node metastasis in PTC showed that age (*p* = 0.002), pathological T-stage (*p* < 0.001) and TPO expression (*p* < 0.001) were significantly associated with recurrence of PTC was significantly associated (Table 1). We used the same approach to explore the predictive value of TPO for PTC recurrence. The analysis showed that T-stage (*p* = 0.01), M-stage (*p* = 0.04) and TPO expression (*p* = 0.008) were significantly associated with the recurrence of PTC (Table 2).

**Table 1:** Binary logistic regression analysis with multiple factors for papillary thyroid carcinoma lymph node metastasis.

Covariates	Exp (B)	95% CI	*p* value
Gender (ref. male)	0.703	0.44–1.121	*p* = 0.139
Age (ref. 11–30)			*p* = 0.002
31–50	2.462	0.032–0.371	
51–70	1.767	0.052–0.586	
71–90	0.81	0.352–1.864	
Pathologic T (ref. T1)			*p* < 0.001
T2	0.109	0.032–0.371	
T3	0.174	0.052–0.586	
T4	0.314	0.096–1.531	
Pathologic M1(ref. M0)	0.722	0.473–1.104	*p* = 0.133
TPO(FPKM)	0.597	0.476–0.748	*p* < 0.001

**Table 2:** Binary logistic regression analysis with multiple factors for papillary thyroid carcinoma recurrence.

Covariates	Exp (B)	95% CI	*p* value
Gender (ref. male)	1.454	0.494–4.28	*p* = 0.496
Age (ref. 11–30)			*p* = 0.736
31–50	0.962	0.16–5.767	
51–70	0.736	0.139–3.904	
71–90	0.481	0.084–2.752	
Pathologic T (ref. T1)			*p* = 0.010
T2	0.487	0.034–6.906	
T3	1.886	0.179–19.823	
T4	1.401	0.144–13.642	
Pathologic N1 (ref. N0)	0.457	0.089–2.35	*p* = 0.2
Pathologic M1 (ref. M0)	0.286	0.108–0.753	*p* = 0.04
TPO (FPKM)	0.509	0.308–0.839	*p* = 0.008

### Validation of TPO expression in PTC tissues from our center and its relationship with lymph node metastasis in patients

**Figure 3:**
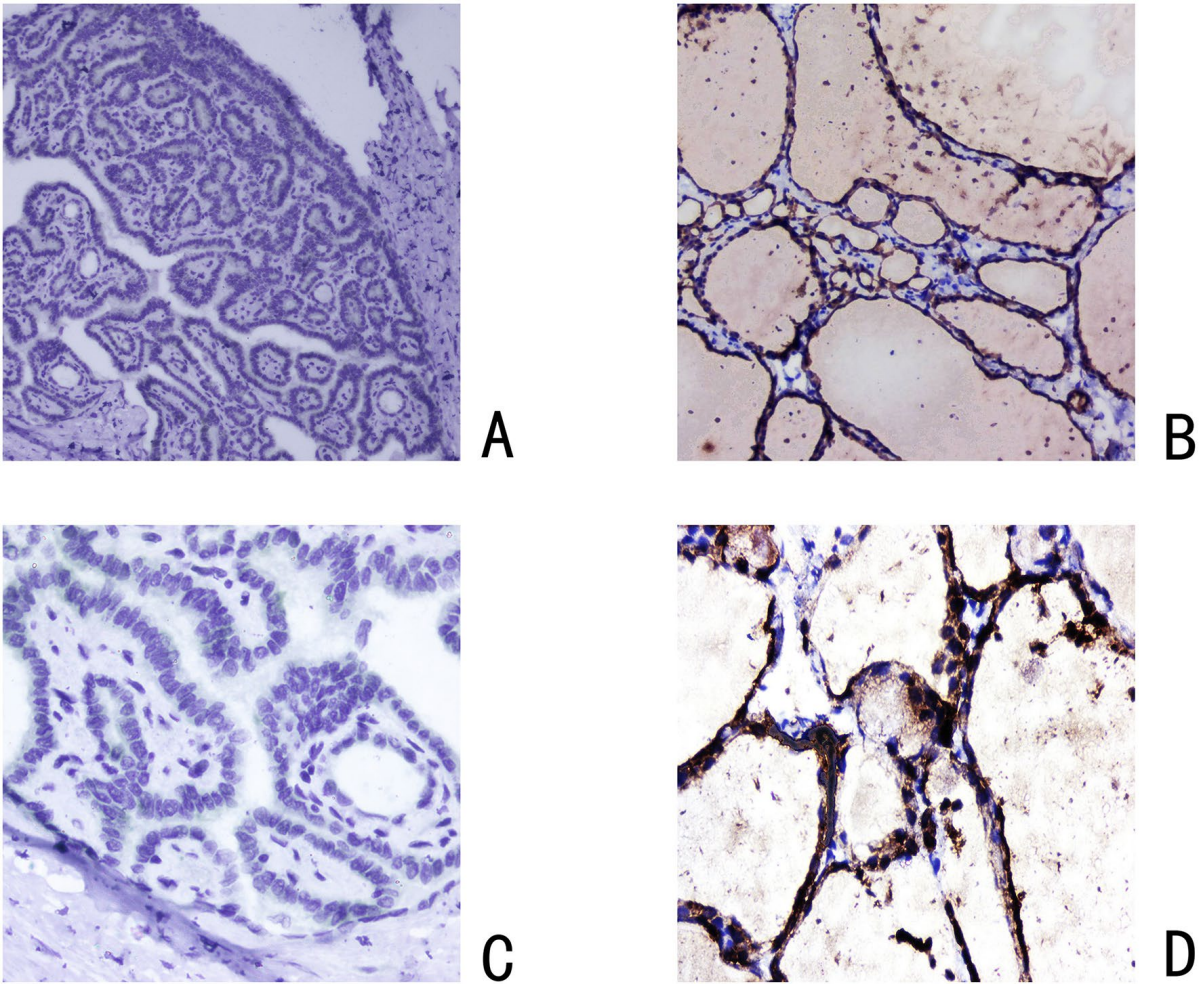
Immunohistochemical results of PTC patient specimens from the First Affiliated Hospital of Kunming Medical University showing (**A**) Negative expression of TPO in PTC tissues (IHC × 100). (**B**) Positive expression of TPO in normal thyroid tissues (IHC × 100). (**C**) Negative expression of TPO in PTC tissue (IHC × 400). (**D**) Positive expression of TPO in normal thyroid tissue (IHC × 400).

To verify the expression of TPO in PTC tissues and its relationship with lymph node metastasis in patients, we selected a total of 230 tumor samples from PTC patients and their clinical data from the Thyroid Disease Clinic of the First Affiliated Hospital of Kunming Medical University for analysis. We verified the expression of TPO in PTC samples by immunohistochemistry (Fig. 3A–D). We found that the expression of TPO in PTC samples was significantly lower than that in normal thyroid tissue. Among our 230 PTC samples, only 36 samples expressed TPO positivity, with an expression rate of 15.7%, whereas their corresponding normal tissues all expressed TPO positivity, with an expression rate of 100%. We then analyzed the relationship between TPO expression and lymph node metastasis in patients by their corresponding clinical data, and the results showed a strong negative correlation between TPO expression and lymph node metastasis in patients (*p* = 0.004) (Table 3). This is consistent with our results analyzed through the TCGA database, demonstrating the role of TPO expression in predicting lymph node metastasis in patients.

**Table 3:** Univariate and multivariate analysis of lymph node metastases in papillary thyroid carcinoma in validation cohort.

Covariates	Univariate analysis		Multivariate analysis		
	X^2^	*p* value	Exp (B)	95% CI	*p* value
Gender (ref. male)	2.172	*p* = 0.141			
Age (ref. 11–30)	13.393	*p* = 0.004			*p* = 0.041
31–50			2.833	0.32–25.085	
51–70			7.219	0.886–58.838	
71–90			5.184	0.566–47.472	
Pathologic T (ref. T1)	27.825	*p* < 0.001			*p* < 0.001
T2			0.096	0.008–1.141	
T3			0.404	0.03–5.377	
T4			2.18	0.03–53.03	
Pathologic M1 (ref. M0)	11.281	*p* = 0.001	0.396	0.11–1.428	*p* = 0.157
TPO (positive) (ref. Negative)	10.725	*p* = 0.001	4.149	1.563–11.017	*p* = 0.004

### Construction of protein–protein interactions network of co-expressed genes and functional annotation and prediction of signaling pathways

**Figure 4:**
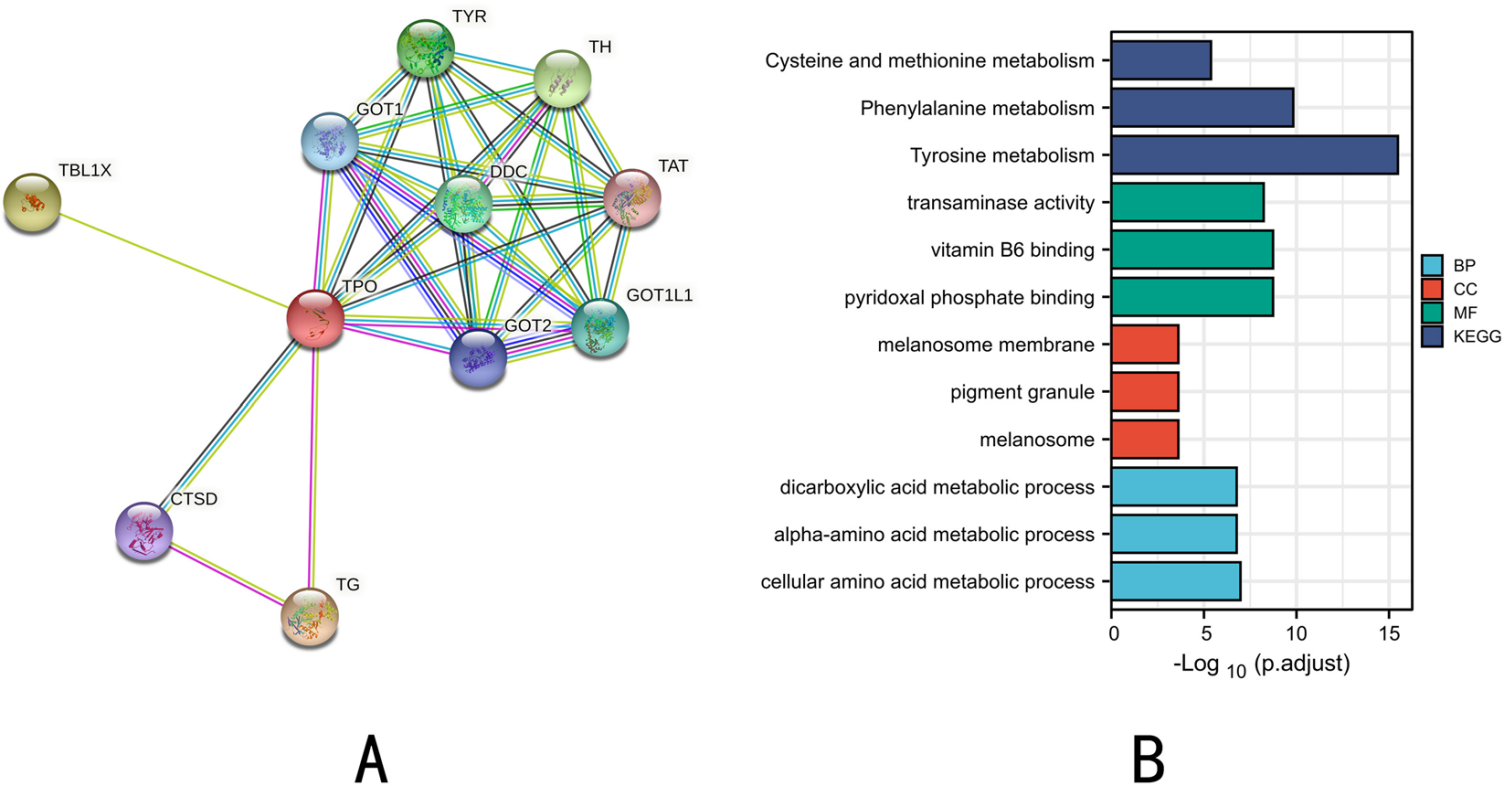
(**A**) Protein–protein interaction networks of TPO protein. (**B**) GO and KEGG enrichment analysis of TPO and its interacting genes.

The single-protein PPI network analysis was performed by the STRING tool. there are 18 edges and 11 nodes in the PPI network (Fig. 4A). The GO and KEGG enrichment analysis of TPO and its interacting genes was performed by the clusterProfiler package (Fig. 4B). Important KEGG pathways are Tyrosine metabolism, Phenylalanine metabolism, and Cysteine and methionine metabolism. Important GO terms enriched for BP are dicarboxylic acid metabolic process, The important GO terms rich in CC are melanosome membrane, pigment granule, and melanosome. Important GO terms for MF-rich are transaminase activity, vitamin B6 binding, and pyridoxal phosphate binding.

### Important genes and pathways acquired by GSEA

**Figure 5:**
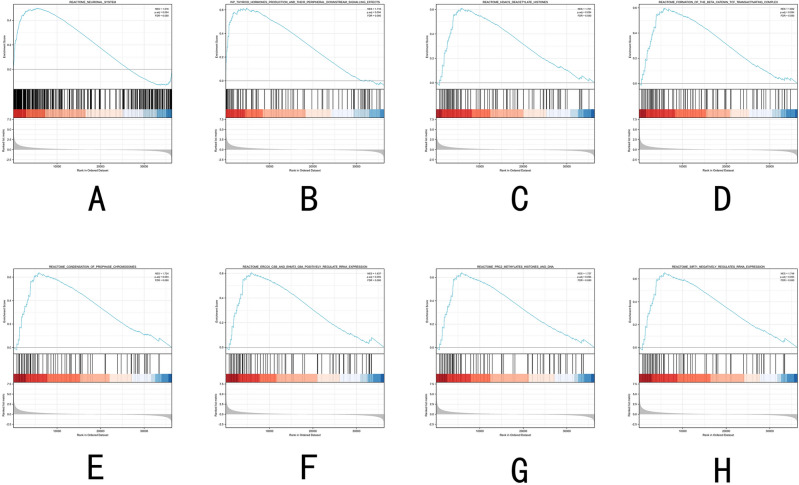
Significant related genes and hallmarks pathways in PTC obtained by GSEA.

To better understand the role of TPO in the development of thyroid cancer, we applied GSEA to analyze the signatures of TPO (Fig. 5A–H). The results showed that the main pathways of TPO include REACTOME_NEURONAL_SYSTEM, WP_THYROID_HORMONES_PRODUCTION_AND_THEIR_PERIPHERAL_DOWNSTREAM_SIGNALLING_EFFECTS, REACTOME_HDACS_DEACETYLATE_HISTONES, REACTOME_FORMATION_OF_THE_BETA_CATENIN_TCF_TRANSACTIVATING_COMPLEX, REACTOME_CONDENSATION_OF_ PROPHASE_CHROMOSOMES, REACTOME_ERCC6_CSB_AND_EHMT2_G9A_POSITIVELY_REGULATE_RRNA_EXPRESSION, REACTOME_PRC2_METHYLATES_HISTONES_AND_ DNA, REACTOME_SIRT1_NEGATIVELY_REGULATES_RRNA_EXPRESSION.

### Analysis of TPO and immune cell correlation

**Figure 6:**
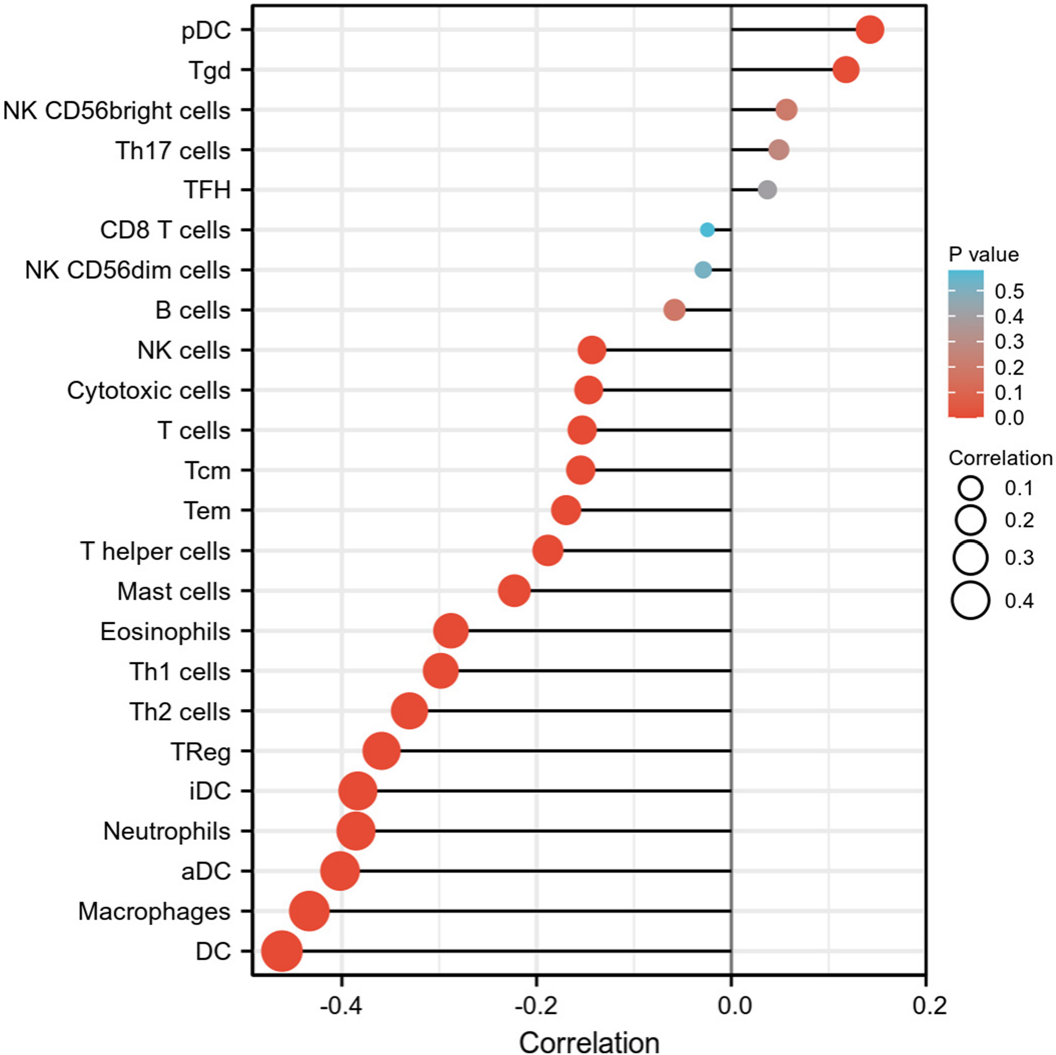
Correlation analysis between TPO and related immune cells in PTC.

We also analyzed the correlation between TPO and PTC immune cells through a database (Fig. 6). The results showed that TPO expression was associated with a variety of immune cells. These included several T cells and their subpopulations, dendritic cells (DC) and their subtypes and two natural killer (NK) cells and their subtypes, as well as neutrophils, mast cells, macrophages, eosinophils and B cells.

The results revealed a strong negative correlation between TPO and dendritic (DC) cells. Meanwhile, TPO had significant negative correlations with macrophages, activated dendritic cells (aDC), neutrophils, immature dendritic cells (iDC), regulatory T (Treg) cells, Th1 cells, and Th2 cells. In addition, there was a positive correlation between TPO and plasmacytoid dendritic cells (pDC).

## Discussion

Although PTC is a malignant tumor with a good prognosis, as a malignant tumor, its metastasis and recurrence still deserve our attention. The main metastasis of PTC is lymph node metastasis in the neck, usually first to the central cervical lymph nodes and then to the lateral cervical lymph nodes^19^. Today, ultrasound is commonly used clinically to assess lymph node metastases. The technique of ultrasound assessment of lymph node metastasis is becoming more sophisticated, but a certain number of missed diagnoses cannot be avoided, which has a great impact on the diagnosis and treatment of patients^20,21^. At the same time, PTC has a certain probability of recurrence^22^. The vast majority of patients with recurrence require re-surgical treatment, which places a financial and physical burden on the patient. Moreover, reoperation also increases the risk of postoperative complications, such as damage to the recurrent laryngeal nerve and temporary or permanent hypoparathyroidism^23^. Therefore, finding factors that predict lymph node metastasis and recurrence of PTC will greatly help in the diagnosis, treatment, and postoperative monitoring of PTC patients.

TPO is a protein widely found in thyroid tissues, and the human gene encoding TPO is located on chromosome 2 and is approximately 150 kb long, consisting of 16 introns and 17 exons. TPO is not only important in thyroid hormone synthesis and maintenance of stable thyroid function, but also plays an important role in the diagnosis and treatment of many thyroid diseases. According to previous reports, TPO is mostly highly expressed in normal thyroid groups and benign thyroid diseases, while it is mostly lowly expressed or absent in papillary thyroid carcinoma^24,25^. Therefore, TPO expression is commonly used clinically to identify the benign and malignant thyroid diseases. This time our study also validated this idea again, in our sample of 230 PTC cases, the positive expression of TPO in PTC tissue was only 15.7%, while it reached 100% in its corresponding normal thyroid tissue. We have observed in our clinic that TPO expression may have a relationship with lymph node metastasis in PTC patients and their disease recurrence.

To find whether the TPO gene and its encoded protein are associated with lymph node metastasis and recurrence in PTC patients, we conducted a study that included a discovery phase and a validation phase. In the discovery phase, we first compared the TPO gene and its proteins in PTC tissues and in thyroid tissues through the TCGA database. We then combined the clinicopathological information of patients in the TCGA database and confirmed that the expression of TPO gene in PTC patients was associated with their lymph node metastasis and recurrence, and the higher the expression of TPO, the lower their lymph node metastasis rate and the lower their recurrence rate. We then selected thyroid samples from 230 PTC patients in our center and recorded their clinicopathological information for validating the relationship between TPO expression and lymph node metastasis and recurrence. Our validation also confirmed that TPO showed low expression in PTC, significantly lower than in normal thyroid tissue, and that TPO expression in PTC tissue was closely associated with lymph node metastasis in patients. Because thyroid cancer has a better prognosis, the sample size in our validation cohort was small and the follow-up period was short, we failed to identify patients with recurrence, and our study failed to compare the differences in recurrence in this group of patients.

To gain more insight into the possible functions of TPO in PTC, we also searched for co-expressed genes of TPO genes and performed enrichment analysis on them. The results showed that the process is mainly related to amino acid metabolism, which may act not only as a substrate but also as a metabolite and metabolic regulator of tumor proliferation^26^. Many existing studies have demonstrated that abnormal amino acid metabolism plays an extremely important role in the development of tumorigenesis^27,28^. According to the results of enrichment analysis, our study found that tyrosine metabolism may be closely related to TPO and its related proteins, according to previous studies, tyrosine metabolism is closely associated with the development of a variety of tumors, such as hepatocellular carcinoma, gastric cancer and esophageal cancer^29,30^. However, there is no report on its relationship with thyroid cancer. In addition, phenylalanine metabolism, cysteine and methionine metabolism, also have a very close relationship with TPO and its related proteins. To understand more specifically the potential role of TPO in thyroid cancer development, GSEA was applied to determine the possible pathways of its enrichment, and the results showed that the high expression of TPO was mainly associated with the regulation of gene expression and signaling pathways related to tumorigenesis development.

Multiple studies have shown that the development of thyroid cancer is closely associated with immune cell infiltration^31^. In PTC, we found that TPO expression was associated with a variety of immune cells. TPO expression had a strong negative correlation with DC cells. A previous study confirmed a close relationship between DC cells, neutrophils and PTC tumor development, and the more DC cells and neutrophils infiltrated, the more aggressive the tumor, the higher percentage of lymph node metastasis and the worse prognosis of PTC patients^32^. In addition, macrophages were also significantly associated with TPO expression. Similarly, macrophages were also closely associated with the development of PTC, and the higher the abundance and proportion of macrophages, the more aggressive the tumor and the higher the rate of lymph node metastasis^31^. Thus, our study reveals a potential role of TPO in PTC immune cell infiltration. These results suggest that TPO plays a crucial role in regulating immune cell infiltration in PTC and its inflammatory response.

There are some limitations of this study. First, in this study, we collected data from patients in our center for validation, but no patients with relapse have been identified in our collection of patients. So the relationship between TPO expression and patient recurrence was not verified in our validation. This may be related to the relatively low recurrence rate of thyroid cancer, the recurrence rate that is well controlled by the existing treatment regimen norms, and also to the short follow-up period of our study. Second, the mechanism of TPO action in PTC is not fully understood, and future studies need to explore the detailed mechanism of TPO in PTC.

## Conclusion

We have investigated that TPO expression may be associated with lymph node metastasis and recurrence in PTC patients through bioinformatics analysis as well as clinical and pathological findings in PTC patients in our center. This study provides new and promising insights for subsequent studies to elucidate the molecular pathogenesis of PTC. At the same time, this may become helpful for our treatment of PTC in the future.

## Data Availability

Transcriptomic and clinical data for all thyroid cancer samples were obtained from the TCGA database (https://portal.gdc.cancer.gov/repository). Expression information of TPO in thyroid gland tissue a data was obtained from the HPA Database https://www.proteinatlas.org/ENSG00000115705-TPO).
